# Implementing cardiovascular disease prevention guidelines to translate evidence-based medicine and shared decision making into general practice: theory-based intervention development, qualitative piloting and quantitative feasibility

**DOI:** 10.1186/s13012-019-0927-x

**Published:** 2019-08-30

**Authors:** Carissa Bonner, Michael Anthony Fajardo, Jenny Doust, Kirsten McCaffery, Lyndal Trevena

**Affiliations:** 1 The University of Sydney, Sydney School of Public Health, ASK-GP Centre of Research Excellence, Rm 128 Edward Ford Building (A27), Sydney, NSW Australia; 2 Bond University, Faculty of Health Sciences & Medicine, ASK-GP Centre of Research Excellence, Robina, QLD Australia

**Keywords:** Cardiovascular disease, Primary care, Risk communication, Risk assessment, Behaviour change, Evidence-based medicine, Shared decision making, Decision aids, Audit and feedback

## Abstract

**Background:**

The use of cardiovascular disease (CVD) prevention guidelines based on absolute risk assessment is poor around the world, including Australia. Behavioural barriers amongst GPs and patients include capability (e.g. difficulty communicating/understanding risk) and motivation (e.g. attitudes towards guidelines/medication). This paper outlines the theory-based development of a website for GP guidelines, and piloting of a new risk calculator/decision aid.

**Methods:**

Stage 1 involved identifying evidence-based solutions using the Behaviour Change Wheel (BCW) framework, informed by previous research involving 400 GPs and 600 patients/consumers. Stage 2 co-developed website content with GPs. Stage 3 piloted a prototype website at a national GP conference. Stage 4 iteratively improved the website based on “think aloud” interviews with GPs and patients. Stage 5 was a feasibility study to evaluate potential efficacy (guidelines-based recommendations for each risk category), acceptability (intended use) and demand (actual use over 1 month) amongst GPs (*n* = 98).

**Results:**

Stage 1 identified GPs as the target for behaviour change; the need for a new risk calculator/decision aid linked to existing audit and feedback training; and online guidelines as a delivery format. Stage 2-4 iteratively improved content and format based on qualitative feedback from GP and patient user testing over three rounds of website development. Stage 5 suggested potential efficacy with improved identification of hypothetical high risk patients (from 26 to 76%) and recommended medication (from 57 to 86%) after viewing the website (*n* = 42), but prescribing to low risk patients remained similar (from 19 to 22%; *n* = 37). Most GPs (89%) indicated they would use the website in the next month, and 72% reported using it again after one month (*n* = 98). Open feedback identified implementation barriers including a need for integration with medical software, low health literacy resources and pre-consultation assessment.

**Conclusions:**

Following a theory-based development process and user co-design, the resulting intervention was acceptable to GPs with high intentions for use, improved identification of patient risk categories and more guidelines-based prescribing intentions for high risk but not low risk patients. The effectiveness of linking the intervention to clinical practice more closely to address implementation barriers will be evaluated in future research.

**Electronic supplementary material:**

The online version of this article (10.1186/s13012-019-0927-x) contains supplementary material, which is available to authorized users.

## Background

### CVD prevention guidelines

Cardiovascular disease prevention guidelines around the world recommend assessing the absolute risk of a heart attack or stroke in the next 5–10 years. This is intended to guide the use of blood pressure and cholesterol-lowering medication for those at “high risk” [1, 2], based on the most predictive risk factors [3]. Reviews show the absolute risk approach can improve clinical management of CVD risk, patients’ risk perception and patients’ preventive intentions, compared to treating hypertension and hyperlipidaemia as separate risk factors [4, 5]. Treatment based on high absolute risk may prevent over-treatment of low-risk patients with an isolated risk factor and under-treatment of high-risk patients with multiple elevated risk factors [6].

### The evidence-practice gap

Despite available guidelines, absolute risk is often not assessed. When it is assessed, it is not necessarily used to guide management decisions [7–10]. A survey of 2000 clinicians across North/South America and Europe found less than half use absolute risk scores regularly. Those who did not use absolute risk were less likely to identify the need for lipid-lowering medication in a hypothetical patient scenario [7]. The consequences are significant. In Australia, 75% of high-risk patients are not receiving recommended medication to prevent death and disability from CVD, and 25% of low risk patients are taking medication they are unlikely to benefit from [11]. This evidence-practice gap is estimated to cost the national health system AU$5.4 billion [12]. Interventions to improve the use of absolute risk assessment in Australia have included pre-consultation risk assessment and integrating risk calculators with medical software [13–15], but these interventions had limited impact on prescribing and have not been translated into clinical practice nationally.

### Behavioural barriers

The Healthy Heart Study aimed to identify and understand behavioural barriers to CVD prevention guidelines in Australia through research with 400 GPs and 600 patients/consumers in 2011–2018 (see Table 1). This project identified barriers not addressed in previous trials, including psychological capability (lack of knowledge about how risk factors relate to medication and lifestyle guidelines, and difficulty understanding/explaining absolute CVD risk); physical opportunity (lack of access to updated evidence and risk communication tools that match Australian guidelines); and reflective motivation (concerns about how to apply guidelines to challenging patient scenarios) [16, 17]. Two strategies have strong evidence to address these issues: audit and feedback can improve knowledge and motivation; and patient decision aids improve risk perception and communication [25, 26]. Audit and feedback programs on this topic were already available to GPs, involving audit of 10 patients with feedback comparing performance to guidelines and peers. There were no available tools to assist GPs with communicating Australian absolute risk guidelines to patients. Systematic reviews of existing online CVD risk communication tools (73 risk calculators and 25 decision aids) found none that met Australian guidelines, used best practice risk communication formats, followed international patient decision aid standards for presenting all management options in a balanced way or met the needs of people with lower health literacy [23, 24].

**Table 1:** Healthy Heart Study findings

	Study description	Implications
1	GP interviews about CVD risk assessment and management [16, 17]	GPs use a range of CVD risk assessment strategies, and identified capability (knowledge, communication) opportunity (access, time) and motivation (habit, concerns about applicability of guidelines to certain patients) as key barriers to absolute risk assessment
2	Patient interviews about CVD risk assessment and management [18]	Patient and GP decision making about CVD risk management is influenced by perceived risk and attitudes rather than calculated absolute risk of a CVD event
3	GP experiment to explore relative influence of absolute risk vs blood pressure/cholesterol on prescribing [19]	Providing an absolute CVD risk assessment is not sufficient to overcome GPs’ tendency to prescribe medication based on blood pressure/cholesterol alone
4	Patient “think aloud” study using heart age calculators [20]	Heart age calculators prompted emotional reactions and consideration of lifestyle changes, but unexpected ‘older’ heart age results were not believable
5	Patient experiment testing heart age versus 5-year absolute CVD risk [21]	Heart age is easier to recall but also inflates risk perception and is less credible than 5-year absolute CVD risk, with no advantage for lifestyle change intentions
6	Patient “think aloud” study using absolute risk calculators [22]	Absolute CVD risk is more meaningful when provided alongside a verbal description of the risk category and graphical displays of intervention effects for both lifestyle and medication
7	Systematic review of existing CVD risk calculators [23]	There were 73 CVD risk calculators available online, but none matched Australian guidelines and they were not suitable for people with lower health literacy
8	Systematic review of CVD decision aids [24]	There were 25 CVD decision aids available online, but none matched Australian guidelines, few presented both lifestyle and medication options in a balanced way, and they were not suitable for people with lower health literacy

### Aim

This study aimed to develop, pilot and evaluate the feasibility of a new online platform for the Australian CVD prevention guidelines that links existing strategies (risk calculator, audit and feedback) with a new patient decision aid, in order to (1) help GPs identify guideline-based recommendations for medication and lifestyle change and (2) communicate this to patients. This paper outlines the results of a theory-based intervention development process, qualitative piloting and quantitative feasibility research for the new combined risk calculator/decision aid component that was not previously available to Australian GPs.

## Methods

The methods involved 5 different stages:

1. Intervention development based on Behaviour Change Wheel process
2. Co-design of content with GPs
3. GP conference feedback on prototype website
4. GP and patient interview feedback on functional website
5. Feasibility study with GPs using final website over 1 month

Ethical approval was obtained via the University of Sydney and Royal Prince Alfred Hospital Human Research Ethics Committees.

### Setting

The project was conducted in Australia in 2017–2018, based on national CVD prevention guidelines released in 2009 (assessment) and 2012 (management) [1, 27]. The guidelines target General Practitioners (GPs) who can be accessed free of charge under the Medicare system. They are based on the 5-year Framingham model of absolute CVD risk, with different recommendations for:

- Low risk (< 10%): no medication with lifestyle change (smoking, diet, exercise) as needed;
- Moderate risk (10–15%): lifestyle change initially, unless extra risk factors are present or lifestyle change is ineffective (in which case medication should be considered); and
- High risk (> 15%): both blood pressure/cholesterol lowering medication and lifestyle change.

### Stage 1: Intervention development based on the Behaviour Change Wheel process

The first stage for this paper was to clearly articulate the problem and solutions in behavioural terms, to address the issues identified in the Healthy Heart Study. We used the Behaviour Change Wheel (BCW) framework because it synthesises multiple health behaviour theories and models to guide the development of rigorous interventions [28]. According to this framework, behaviour can be attributed to three determinants of behaviour: opportunity (physical and social environment), capability (physical and psychological ability) and motivation (automatic and reflective mechanisms). The framework outlines a process to identify the most important behavioural barriers, the best target population for behaviour change, evidence-based behaviour change techniques and the most feasible delivery mode.

### Stage 2: Co-design of content with GPs

The website content was co-developed with GPs via the ‘Ask Share Know: Rapid Evidence for General Practice Decisions: (ASK-GP) Centre of Research Excellence Clinical Laboratory.’ This includes a ‘knowledge broker’ service that provides evidence-based resources for GPs to discuss at small group meetings, which were audio-recorded to supplement field notes (CB and MF). Two GP groups were included as they were running discussions at the time of the study. Qualitative data were obtained via field notes to document any suggested changes or problems identified by users as part of the co-design process, with audio recordings used to clarify field notes if needed. A summary of each group discussion was written, and this document was scanned to identify changes to be made for the next website version. No formal qualitative analyses were conducted.

### Stage 3: GP conference feedback on prototype website

The website prototype was piloted at an Australian national conference for General Practitioners in 2017 (GP17) via a presentation and question/answer session (CB) and a tablet placed in an exhibition room stall (CB). Conference data included notes from verbal discussion with stall visitors and a brief written feedback form from attendees with open responses and an overall acceptability rating out of 10.

### Stage 4: GP and patient interview feedback on functional website

After the GP17 conference, the functional website was developed iteratively based on semi-structured ‘think aloud’ user interviews [29] to improve acceptability. A more detailed Framework Analysis had been conducted in 1 GP interview study and 2 patient think aloud studies prior to this stage (see Table 1), so thematic analysis for this stage was limited to notes taken during the interviews (CB and MF) and from audio recordings, to identify areas to improve. Interviews were conducted at the University of Sydney, via Skype or at the participants’ residence/workplace, and were audio-recorded to supplement field notes on intervention features to improve. This involved a concurrent and retrospective verbal protocol where users were asked to think aloud as they used the website, followed by prompting for feedback by the interviewer (CB) [20].

### Stage 5: Feasibility study with GPs using final website over 1 month

A feasibility study to assess acceptability (intended use after initial viewing), demand (actual use after 1 month) and potential efficacy (improved knowledge of recommended interventions for each risk category as per guidelines) [30] was conducted for the final website. A pre-post design was used to maximise feedback from ~ 100 end users within the project budget, with oversampling at baseline (*n* = 123) to achieve adequate numbers at follow-up (*n* = 98). GPs were recruited anonymously via an independent recruitment company, which could not provide identifying information about GPs; therefore, it is unknown whether (if any) overlap occurred with the GPs participating in the qualitative stages. The 10-min baseline survey is provided in Additional file 2 and included use of guidelines and absolute CVD risk calculators; self-efficacy for assessing and communicating absolute CVD risk [31]; testing one of 9 hypothetical patients (3 each from low, moderate and high risk categories as per Australian guidelines) with the website to explore whether recommendations matched risk category guidelines for challenging cases [16, 17]; intended use of website features over the next month; open feedback; and demographics. The 4-min follow-up survey was sent in 3 batches 1 month after completion of the baseline survey to standardise the follow-up period (range 4–6 weeks). It repeated the same questions without the hypothetical patient testing or demographics, with self-reported website usage assessed for the previous month. GPs participated anonymously via a specialist recruitment company and received $40 along with access to a login and pre-filled form to apply for continuing professional development points if they chose to complete the self-directed audit and feedback component of the website. Descriptive analyses and confidence interval calculations were conducted using Microsoft Excel, and inferential analyses were conducted using SPSS v25. Chi-squared tests and paired *t* tests were used to compare categorical data and continuous data, respectively, between baseline and follow-up. Only the final sample of 98 was used in the analysis.

## Results

### Stage 1: Intervention development based on Behaviour Change Wheel process

**Fig. 1:**
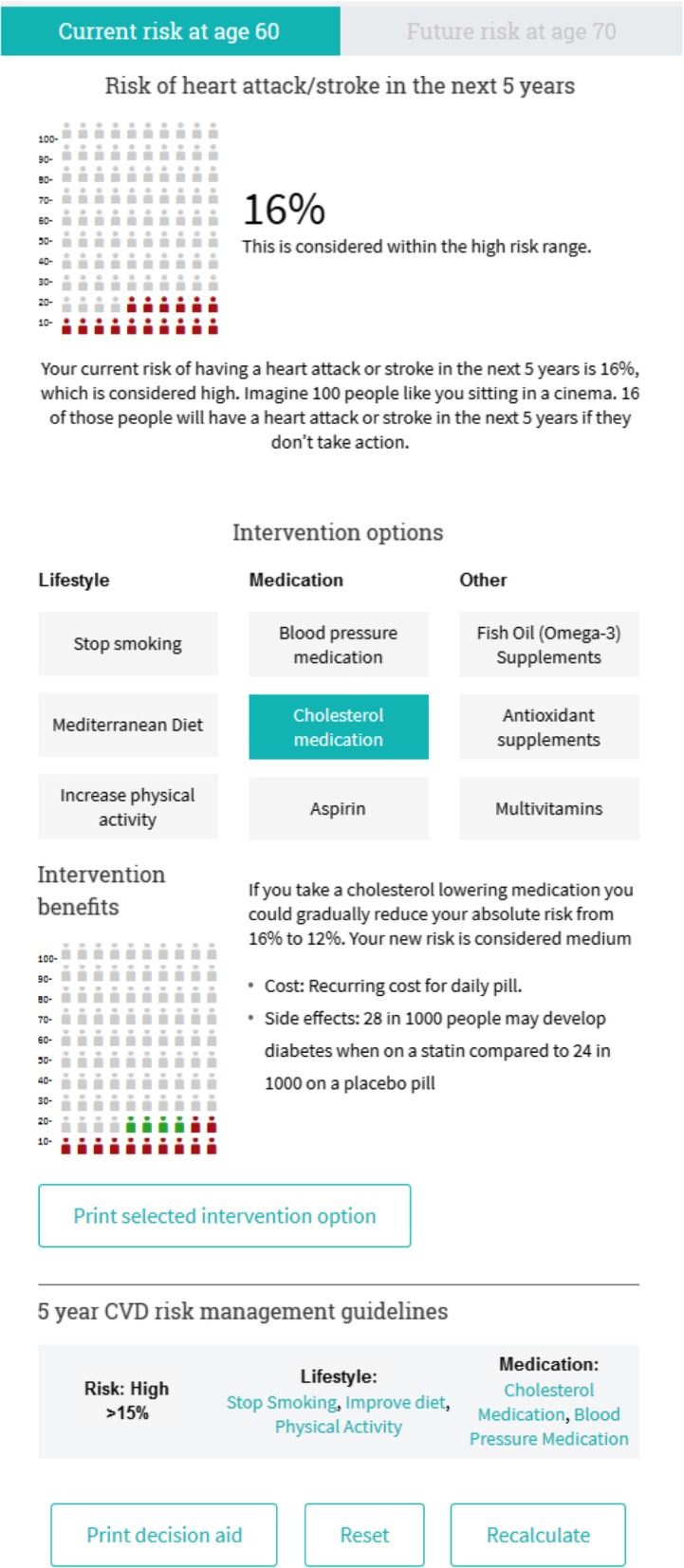
Patient risk calculator and decision aid

**Fig. 2:**
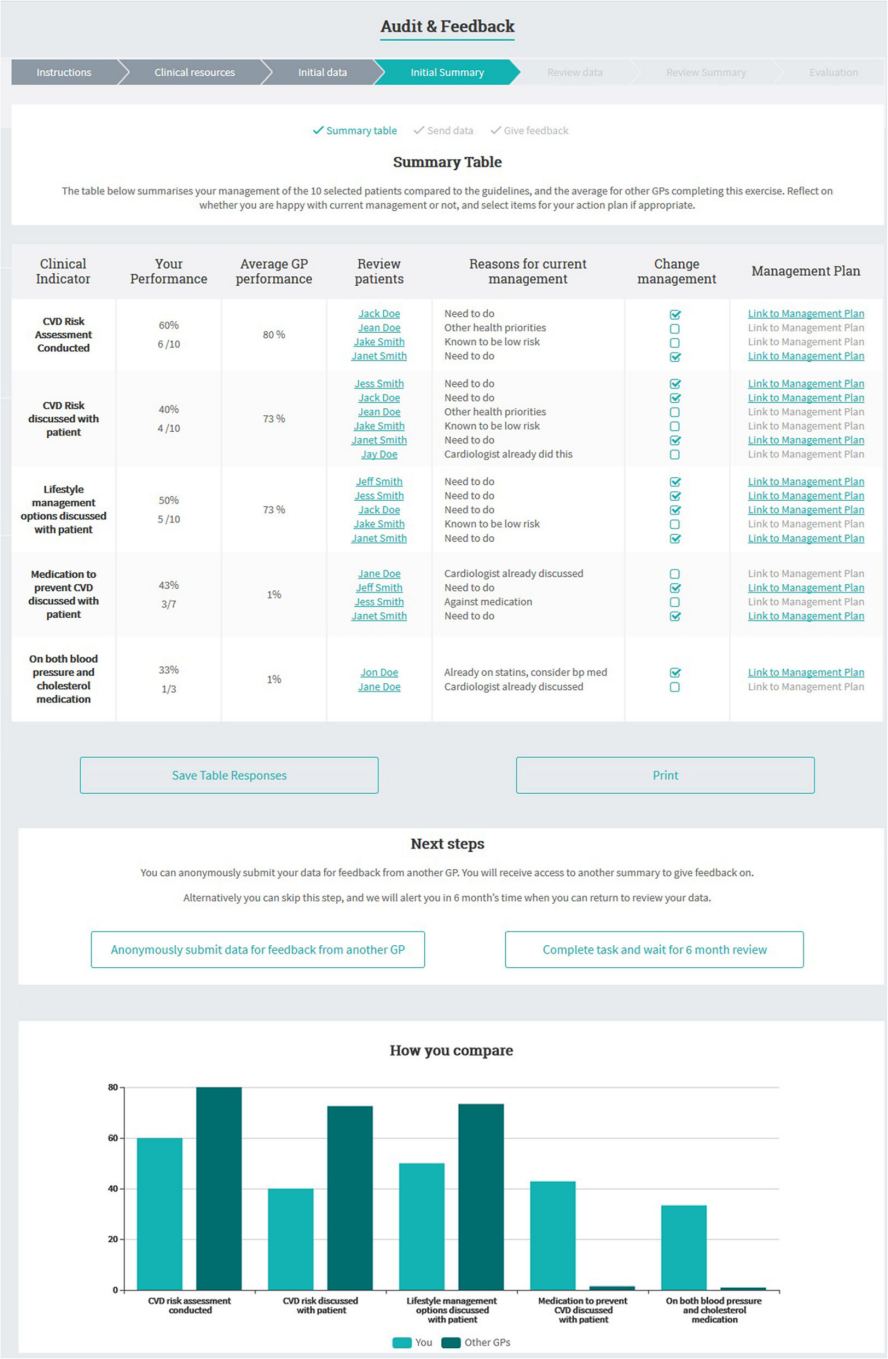
GP audit and feedback exercise

**Fig. 3:**
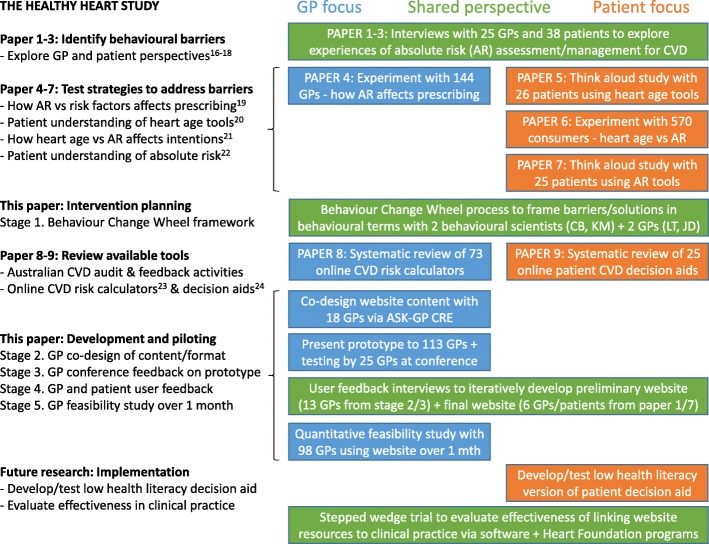
Summary of intervention development and testing

Completing the Behaviour Change Wheel process (summarised in Table 2) identified the need to develop a new tool for GPs to use with their patients in consultations, with the following key features (see Figs. 1, 2 and 3 for screenshots of main features, and Additional files 1 and 2 for TIDIER checklist and more detailed intervention content):

1. Interactive CVD risk calculator that combines CVD risk assessment and management algorithms to help GPs identify risk category guidelines [1, 27], based on best practice risk communication principles and patient perceptions of existing CVD risk calculators [20, 22, 32];
2. Personalised patient decision aid that shows the effect of different medication (blood pressure, cholesterol, aspirin), lifestyle (smoking, diet, exercise) and supplement (antioxidants, omega-3, multivitamins) interventions on individual CVD risk to help GPs discuss the benefits and harms of different options [26], based on updated evidence reviews and International Patient Decision Aid Standards to support shared decision making [33]; and
3. Self-directed audit & feedback including cases that GPs find challenging for CVD risk assessment and communication [16, 17], and comparison of management to guidelines [27], using evidence-based behaviour change techniques [34], based on existing audit and feedback tools familiar to GPs (involving audit of 10 patients with feedback comparing performance to guidelines and peers).

**Table 2:** Summary of conclusions from the Behaviour Change Wheel framework process

Intervention functions	Behavioural components served by intervention functions	Behaviour change techniques (BCTs) to deliver intervention functions	Policy categories through which BCTs can be delivered	Intervention strategy
• Education • Training • Persuasion	• Psychological capability (understanding role of risk factors, risk communication) [16, 17] • Physical opportunity (access to updated evidence on risk/benefit in line with Australian guidelines) [16, 23, 24] • Reflective motivation (attitude towards using guidelines for perceived low/high risk cases) [16]	• Information about health consequences • Feedback on behaviour • Instruction on how to perform a behaviour • Action planning • Social comparison	• Guidelines	Online version of guidelines to enable national access, linked to: • 5-year risk calculator that integrates assessment and management guidelines including clear role of risk factors • Updated evidence on benefits and harms for medication + lifestyle options • Decision aid for patients to improve communication • Hypothetical patient cases with feedback as part of audit and feedback training exercise [based on existing resources]

In terms of the BCW framework [28], the intervention targets GPs during consultations and uses education, training and persuasion functions to address psychological capability (decision aid to improve knowledge of applicable guidelines and communication skills; supported by audit and feedback), physical opportunity (by providing the first CVD prevention decision aid based on Australian guidelines and updated evidence) and reflective motivation (by addressing GP concerns through case studies that show how absolute risk can be assessed and communicated; linked to decision aid and part of audit and feedback). It uses multiple behaviour change techniques (information about health consequences, feedback on behaviour, instruction on how to perform a behaviour, action planning, social comparison) delivered via the policy category of guidelines, using an online format for wide accessibility and flexible/sustainable implementation, see Table 2.

### Stage 2–4: Qualitative piloting of content, prototype website and functional website

Table 3 shows how iterative user feedback led to changes to the website over stages 2–4.

**Table 3:** Website development based on iterative user feedback

Development stage	Example user feedback	Major changes made
Stage 1: Intervention development based on Behaviour Change Wheel process	Healthy Heart Study GP interview: ‘The calculator of course doesn’t include certain factors…if someone does do a lot of exercise I would…think their risk is probably lower.’ [16] Healthy Heart Study patient interview: ‘The visual presentation of the result…because it’s a picture instead of numeric, I think I’ll take more interest…when you see red and green…that seems to have more of a impact on me, you know…the numbers don’t, you know?’ [22]	• Develop new risk calculator to more clearly explain risk factor roles in assessment versus management guidelines (psychological capability), supported by links to existing audit and feedback strategies (reflective motivation) • Link risk calculator to patient decision aid with colour coded icon arrays to help GPs explain probability of CVD event to patients (psychological capability) and access up-to-date intervention effects on their risk (physical opportunity)
Stage 2: Co-design of content with GPs	GP focus group: “Is it possible you need to quote something along the lines of ‘no evidence regarding dose or exact dose’ or something? Cos patients ask you a lot of ‘how much should I take?’… It could be good with a bit of extra information and a little bit more about the doses and the side effects and costs and so on”	• Less statistical information and more practical issues for GP evidence summaries • Include complementary and alternative medicine options to show lack of effect on CVD outcomes
Stage 3: GP conference feedback on prototype website	GP conference feedback: ‘Improved diet’ is very vague and after all, the benefit is only with the Mediterranean diet and has not been shown with other ‘improved diets’. GP interview: ‘So are there explanations…for alcohol, you want to put what moderate means...it’s not going to calculate BMI for you?’	• Rewording risk factors and interventions to be clearer • Automatic calculation of body mass index and risky drinking • Changed icon array shades to cater to vision impairments and black/white printing
Stage 4a: GP and patient interview feedback on functional website	GP interview: ‘It’s directed towards the risk factor they’ve actually identified?... I think this is really good the summary… it’s really comprehensive…let’s not waste any time talking at length about smoking if you’re not even considering it…whereas what about your diet… oh yes I’m keen to know about that’ Patient interview: ‘With the button where it says print…it automatically comes up with the print page ahead of viewing it, so maybe it’s better to view it first and have the option to print later so you don’t have to print it’	• Add print button for 2 page summary of single intervention selected by GP • View decision aid information in separate tab before printing • Change summary table in full 9 option decision aid to more clearly show effects on risk
Stage 4b: GP and patient interview feedback on final website at www.auscvdrisk.com.au	GP interview: ‘The patient would be given an ipad…give it to the nurse or hand it back to reception…doing the AUSDIAB [diabetes risk assessment] at reception was really good…enter it on the patient file…if it was high then I would need know’ Patient interview: ‘I have to talk to the doctor about the cholesterol lowering medication, and blood pressure and aspirin…I can take this [decision aid] with me next time I go to see her’	• No further changes made to GP website or linked resources • Implementation suggestions still need to be addressed: 1. Auto-population of risk factors from patients’ electronic record; 2. Low health literacy version of decision aid; 3. Pre-consultation access to risk calculator/decision aid
Stage 5: Feasibility study with GPs using final website over 1 month	GP open response comments reflecting key implementation issues: ‘If it could be somehow linked to practice software so I remember to do it and the values are prefilled that would be ideal.’ ‘Needs to have some in different languages to show people outcomes for those with poor English understanding.’ ‘More time to be scheduled to counsel patients on lifestyle modification and CVD risk calculator use.’	• Feedback generally positive with some contrasting views on format preferences • Confirmed implementation issues identified in stage 4b: 1. Auto-population of risk factors from patients’ electronic record 2. Low health literacy version of decision aid; 3. Pre-consultation access to risk calculator/decision aid

### Stage 2: Co-design of content with GPs

The website content and format was discussed with two practices involved in the ASK-GP CRE (*n* = 18 GPs), including feedback on examples of evidence summaries, decision aids and CVD risk calculators. Changes were made to the evidence summaries to incorporate more practical issues such as cost and inconvenience to patients. GPs asked about complementary and alternative medicine options (fish oil, antioxidants and multivitamins), which were subsequently included in the risk calculator/decision aid even though they had no effect on CVD outcomes, as the GPs felt it would be helpful to show this to patients in order to direct them to more effective options.

### Stage 3: GP conference feedback on prototype website

The prototype website was demonstrated at the national GP17 conference in October 2017 via a 30-min presentation with a question/answer session (attended by *n* = 113 delegates) and a conference stall where 25 GPs tested the prototype website on a tablet, of which 16 completed a written feedback form. Verbal and written feedback was positive, with an average 8.4/10 overall acceptability rating and comments such as: “So user friendly, takes relevant clinical data into account, very comprehensive recommendations which are patient as well as doctor friendly”.

### Stage 4: GP and patient interview feedback on functional website

In-depth user feedback was obtained from 7 GPs via ASK-GP CRE groups and interested GP17 conference attendees, using semi-structured “think aloud” interviews while using the preliminary website. Suggestions include risk factor and intervention wording changes, automatic calculation of risky drinking and body mass index, and changes to the visual presentation of the risk result to enable black and white printing. These changes were incorporated into the website before further testing with 3 new GPs and 9 patients. Another round of changes was made for the final website halfway through this testing, including a two-page summary of a single intervention as well as the full decision aid. No major content issues were identified in final website testing, but there were some suggestions for implementation to improve accessibility: linking the risk calculator to GP practice software, involving practice nurses and creating a patient/consumer version of the website that is easier to understand without GP consultation. These issues could not be addressed within the project budget for intervention development. The final website is available at www.auscvdrisk.com.au.

### Stage 5: Feasibility study with GPs using final website over 1 month

Tables 4 and 5 provide an overview of the sample and descriptive capability results before and after using the risk calculator/decision aid component of the website.

**Table 4:** Feasibility study participant characteristics

Characteristics	Final sample (*n* = 98)
Age (mean)	52.6 years (SD 8.57)
Experience as GP (mean)	28.0 years (SD 8.98)
Gender	
Male	67 (68%)
Female	30 (31%)
Other/prefer not to say	1 (1%)
Australian state/territory	
New South Wales	31 (32%)
Victoria	28 (29%)
Queensland	22 (22%)
Western Australia	4 (4%)
Tasmania	2 (2%)
Northern Territory	1 (1%)
Australian Capital Territory	1 (1%)

**Table 5:** Correct risk category and medication recommendation before and after using the website

Selected case	Before using risk calculator	After using risk calculator
Low risk (*n* = 37)	Correct risk category: 70%	Correct risk category: 87%
	Likely/very likely to prescribe any meds: 19%	Blood pressure med recommended: 22%
		Cholesterol med recommended: 22%
Moderate risk (*n* = 19)	Correct risk category: 58%	Correct risk category: 90%
	Likely/very likely to prescribe any meds: 74%	Blood pressure med recommended: 32%
		Cholesterol med recommended: 42%
High risk (*n* = 42)	Correct risk category: 26%	Correct risk category: 76%
	Likely/very likely to prescribe any meds: 57%	Blood pressure med recommended: 71%
		Cholesterol med recommended: 83%

Note: see Additional file 1 for details of the 9 hypothetical patient cases (3 per risk category; randomised order in survey and also presented in the audit and feedback section of website; developed from Healthy Heart Study GP interviews that identified situations where absolute risk assessment/communication is most challenging [16, 17])

#### Sample description

The participant characteristics are summarised in Table 4. At baseline, almost all (95%, *n* = 93) reported using absolute CVD risk calculators, most commonly risk calculators within practice software (Best Practice 35%, *n* = 34; Medical Director 21%, *n* = 21) and the National Vascular Disease Prevention Alliance website (www.cvdcheck.org.au; 32%, *n* = 31). A lower proportion (72%, *n* = 71) of GPs had seen the national CVD prevention guidelines.

#### Potential efficacy (guideline-based recommendation for risk category)

**Fig. 4:**
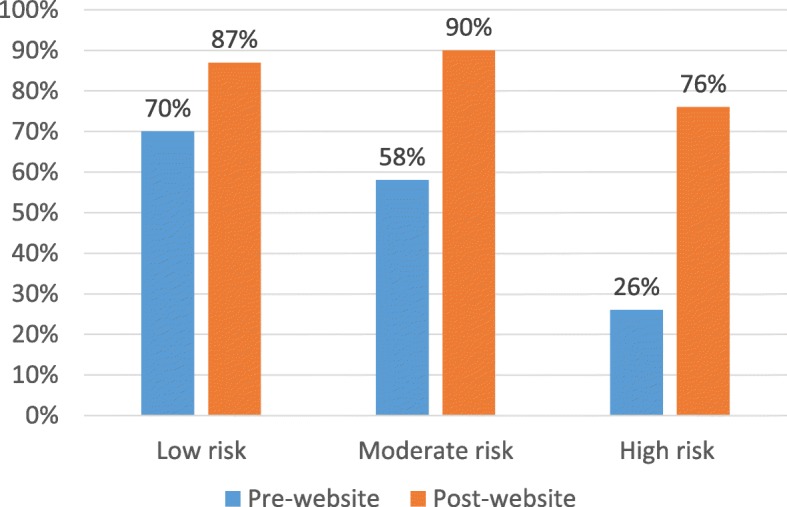
Correct identification of risk category for low, moderate and high risk patient cases

At baseline, using the new decision aid at www.auscvdrisk.com.au with a self-selected hypothetical patient significantly increased identification of the correct risk category for low risk cases by 16% (95% CI 0 to 32%), moderate risk cases by 32% (95% CI 6 to 57%) and high risk cases by 50% (95% CI 35 to 65%); and increased identification of blood pressure and/or cholesterol medication as a recommendation for high risk (see Fig. 4 and Table 5). From 42 GPs who selected a high-risk case, 57% (*n* = 24) were likely/very likely to prescribe medication. After using the risk calculator, 86% (*n* = 36) indicated blood pressure or cholesterol medication were recommended, and 69% (*n* = 29) indicated both were recommended. From 37 GPs who selected a low-risk case, prescribing was similar before (19%, *n* = 7) and after (22%, *n* = 8) using the risk calculator.

#### Acceptability (intended use)

At baseline, most GPs (88%, *n* = 86) intended to use the website (at least 1 feature) over the next month.

#### Demand (actual use over 1 month)

At 1-month follow-up, most GPs (73%, *n* = 72) reported using the website (at least 1 feature) in the last month. This included interactive risk calculator features (73%, *n* = 72), printed decision aid features (67%, *n* = 66) and guideline features (52%, *n* = 51).

#### Other outcomes

At 1-month follow-up, there were no significant pre-post differences in reported use of absolute risk assessments; self-efficacy in conducting an absolute CVD risk assessment; or self-efficacy in explaining absolute CVD risk.

#### Open feedback

In open feedback, improvements were suggested by 44% (*n* = 43) and 40% (*n* = 39) of GPs at baseline and follow-up, respectively. Of all suggested improvements at baseline, improving access (e.g. via Practice Software integration) was most common (48%, *n* = 21) followed by formatting changes (29%, *n* = 13; e.g. colour and font) and content changes (23%, *n* = 10; e.g. more instructions, different website design features, additional risk factor/effect estimates beyond the scope of the guidelines). Similarly, of all suggested improvements at follow-up, improving formatting was most common (58%, *n* = 23) followed by improving access (40%, *n* = 41) and content changes (20%, *n* = 8).

## Discussion

This paper outlines a rigorous theory-based process to develop an intervention to improve the use of guidelines for CVD prevention in Australia, aiming to address GP barriers. It draws on the large literature supporting the use of audit and feedback to change GP prescribing behaviour and use of guidelines, and patient decision aids to improve doctor-patient communication and understanding of risk [25, 26]. It also adds to the growing use of the Behaviour Change Wheel framework for developing public health interventions to diagnose and target behavioural barriers to public health [28]. From the Behaviour Change Wheel Framework, the education, training and persuasion functions to address psychological capability, physical opportunity and reflective motivation components collectively increased the capacity for GPs to correctly identify CVD risk categories for several scenarios. Improvement in intentions to prescribe to high-risk patients was also observed, although there was little change to prescribing to low-risk patients. Use over 1 month and intentions for future use were high suggesting acceptability to end users.

However, we know that both evidence-based medicine and shared decision making face many barriers more broadly, including environmental and system level issues [35]. The pilot findings suggest several directions for implementation that have already been trialled in the Australian primary care context, which may be effective if combined with the existing intervention. Specifically, there are three suggestions from GPs and patients in this study that still need to be addressed (see Table 1):

1. *Pre-consultation access to risk calculator/decision aid*: Both GPs and patients suggested that the risk assessment and decision aid could be accessed prior to a consultation before further discussion, ideally with the support of practice nurses and a more consumer-friendly interface. This method has been trialled previously in Australia for CVD risk assessment using a waiting room method [15], but without the additional support of a risk calculator/decision aid that addressed capability barriers around understanding and communication of risk models. Existing risk calculators did not explain the role of assessment vs management factors, and no decision aids were available that matched 5-year Australian CVD risk guidelines.
2. *Auto-population of risk factors from patients’ electronic record*: This was a suggestion from GPs, who wanted to save time by having the risk assessment linked to patients’ recorded risk factors in medical software. This approach has been trialled previously in Australia [13, 14], but access to the risk calculator was restricted to license holders and did not have the additional support of a decision aid to address communication of risk models. Existing tools were inaccessible or did not match Australian guidelines.
3. *Low health literacy version of decision aid* to meet the needs of people with inadequate skills to access, understand and act on health information. This was suggested by GPs working with diverse communities, who felt the full decision aid with 9 options would be too much information for many of their patients. Low health literacy decision aids have been developed and trialled in Australia using a “universal precautions” approach to health-literate design, but not for CVD prevention [36].

The next stage of the project will use a combination of the above strategies to support the implementation and evaluation of the intervention in clinical practice, as well as aligning with national Heart Foundation programs that aim to improve the use of absolute CVD risk assessment and communication about management options with patients in primary care. The implementation of tools to support shared decision making and health literacy is timely in Australia, with recent policy changes explicitly supporting these two areas [37]. Internationally, there have been calls to use shared decision making in relation to changing CVD prevention guidelines in the UK and US, given lower medication thresholds that may result in many previously ‘low risk’ patients taking medication for very small benefits in risk reduction [38–40]. Additional strategies may be needed to address the overtreatment of low risk patients, since little change was observed in this study.

### Implications for implementation science

Our results highlight a theoretical issue regarding the difference between implementation (prescribing for high risk) and de-implementation (not prescribing for low risk) [41]. While addressing capability barriers to guidelines appeared to increase intentions for prescribing medication to high-risk patients, there was no improvement in unnecessary prescribing for low risk patients. A recent synthesis of behaviour change theory suggests that behaviour substitution may be needed to address the latter situation, but there is little guidance in the literature for how to select such a behaviour at present [41]. More broadly, this project provides a model to other intervention developers for how to apply the theory-based Behaviour Change Wheel framework to identify behavioural barriers to the use of guidelines [28], and co-design an evidence-based intervention with end users to address these barriers. In particular, it illustrates the value of an extensive qualitative evaluation to understand behavioural barriers amongst different targets (GPs and patients) and testing different strategies/formats *before* trialling an evidence-based intervention, as previous trials in this context had little impact on prescribing and failed to incorporate important knowledge (confusion about the role of different risk factors) and capability (doctor-patient risk communication) issues. The iterative co-design process for website development shows how GP and patient feedback can be incorporated into intervention design, but the timeframe required for this process meant that the qualitative analysis was pragmatic rather than formally thematic. Future intervention designers may benefit from: (1) considering implementation and de-implementation components separately as different behavioural strategies may be needed and (2) long-term planning to allow time for detailed exploration of behavioural barriers initially and more in-depth analysis of purposively sampled user feedback during development.

### Strengths and limitations

This paper highlights the usefulness of mixed qualitative and quantitative methods to understand the context and behavioural barriers to a public health issue, and an iterative user feedback process to develop the intervention. The resulting intervention is evidence-based and acceptable to users in Australia, but needs further testing to evaluate its efficacy and use in clinical practice. The main limitations are (1) the use of pragmatic qualitative analysis methods (i.e. documenting issues in field notes/summary documents to facilitate quick feedback to website developers rather than formal thematic analysis); (2) the use of a pre-post design to maximise end user feedback, rather than a randomised trial design; and (3) the use of GP self-report rather than GP-patient interactions within a consultation. Future research will focus on addressing these limitations and the implementation suggestions identified in the pilot study.

## Conclusions

Following a theory-based development process and user co-design, the resulting intervention was acceptable to GPs with high intentions for use, improved identification of patient risk categories and some improvement in intended prescribing for high-risk but not low-risk patients. The effectiveness of linking the intervention to clinical practice more closely to address implementation barriers will be evaluated in future research.

## Additional files

Additional file 1: Components of the Australian CVD guidelines intervention. (DOCX 2701 kb)

Additional file 2: GP survey about cardiovascular disease (CVD) prevention guidelines. (PDF 60 kb)

## Data Availability

The datasets used and/or analysed during the current study are available from the corresponding author on reasonable request.

## Abbreviations

ASK-GP: Ask, Share, Know, Rapid Evidence Reviews for General Practice Decisions
BCW: Behaviour Change Wheel
CVD: Cardiovascular disease
GP: General Practitioner
UK: United Kingdom
US: United States
