# Supplementary material for: Implementing cardiovascular disease prevention guidelines to translate evidence-based medicine and shared decision making into general practice: theory-based intervention development, qualitative piloting and quantitative feasibility
Source: Implement Sci. 2019 Aug 30;14:86. doi: 10.1186/s13012-019-0927-x (PMC6716813; doi:10.1186/s13012-019-0927-x)

**Components of the Australian CVD guidelines intervention (**[**www.auscvdrisk.com.au**](http://www.auscvdrisk.com.au)**)**

An overview of key features is listed below, with more details and screenshots on the following pages.

1. **Summary of Australian guidelines** for CVD risk assessment (2009) and management (2012) for low, moderate and high risk patients.
2. **Updated evidence summaries** based on rapid reviews for high quality studies on lifestyle and medication interventions since 2012.
3. **Risk calculator** based on 5 year Framingham equation and Australian guidelines for clinically determined high risk, based on best practice risk communication principles.
4. **Interactive decision aid** that can be printed as a full decision aid for 9 intervention options based on International Patient Decision Aid Standards, or a 2 page summary for a single intervention.
5. **Audit and feedback exercise** including video demonstration, interactive cases, automated feedback on management of 10 patients compared to guidelines, action planning and peer discussion/comparison.


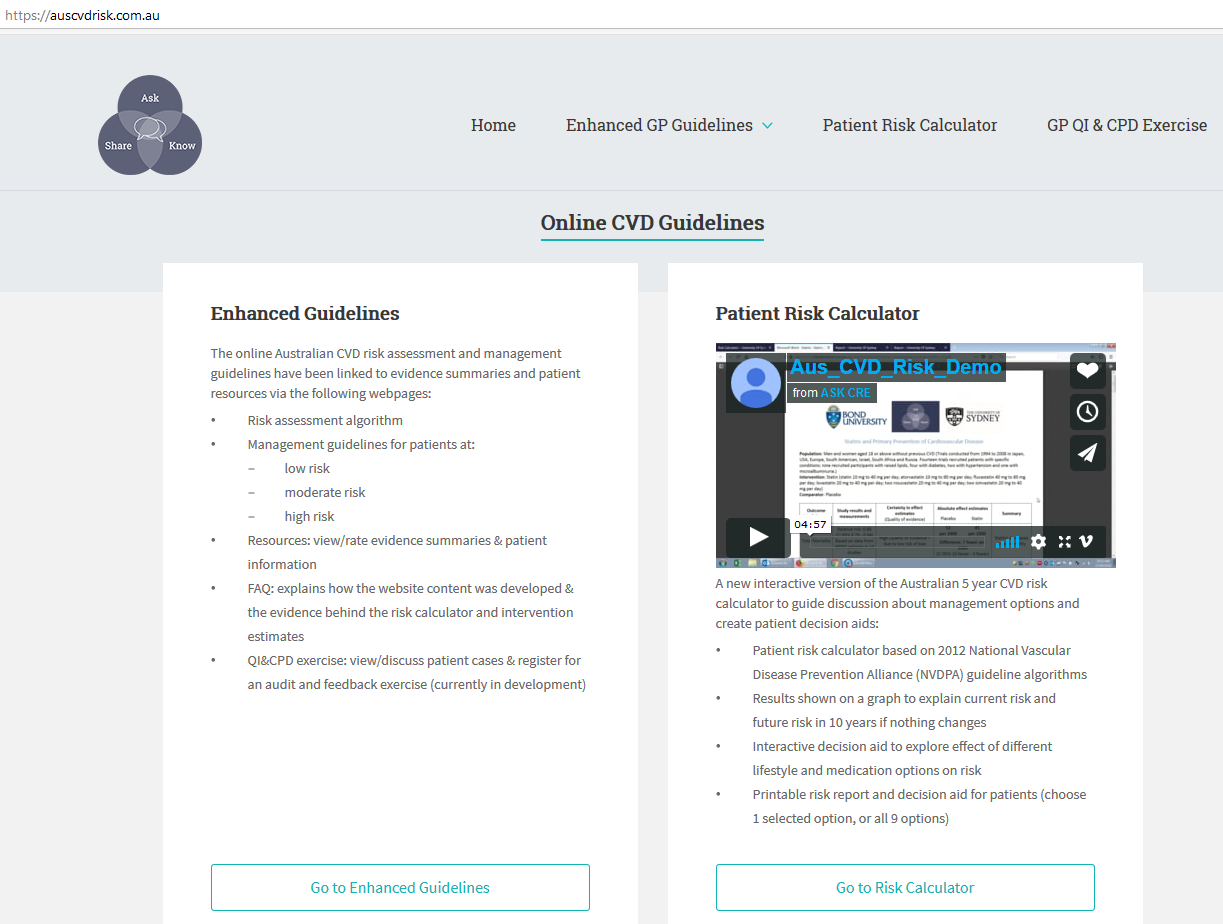


1. **Summary of the Australian CVD prevention guidelines**

The Australian guidelines for 5 year absolute CVD risk assessment (2009) and management for low (<10%), moderate (10-15%) and high (>15%) risk (2012) were used as the basis for the website content and algorithms. Below is an example of the summary for high risk patient management.

***Australian guidelines: example summary flowchart for high risk patient management, including links to updated evidence summaries (see feature 2)***


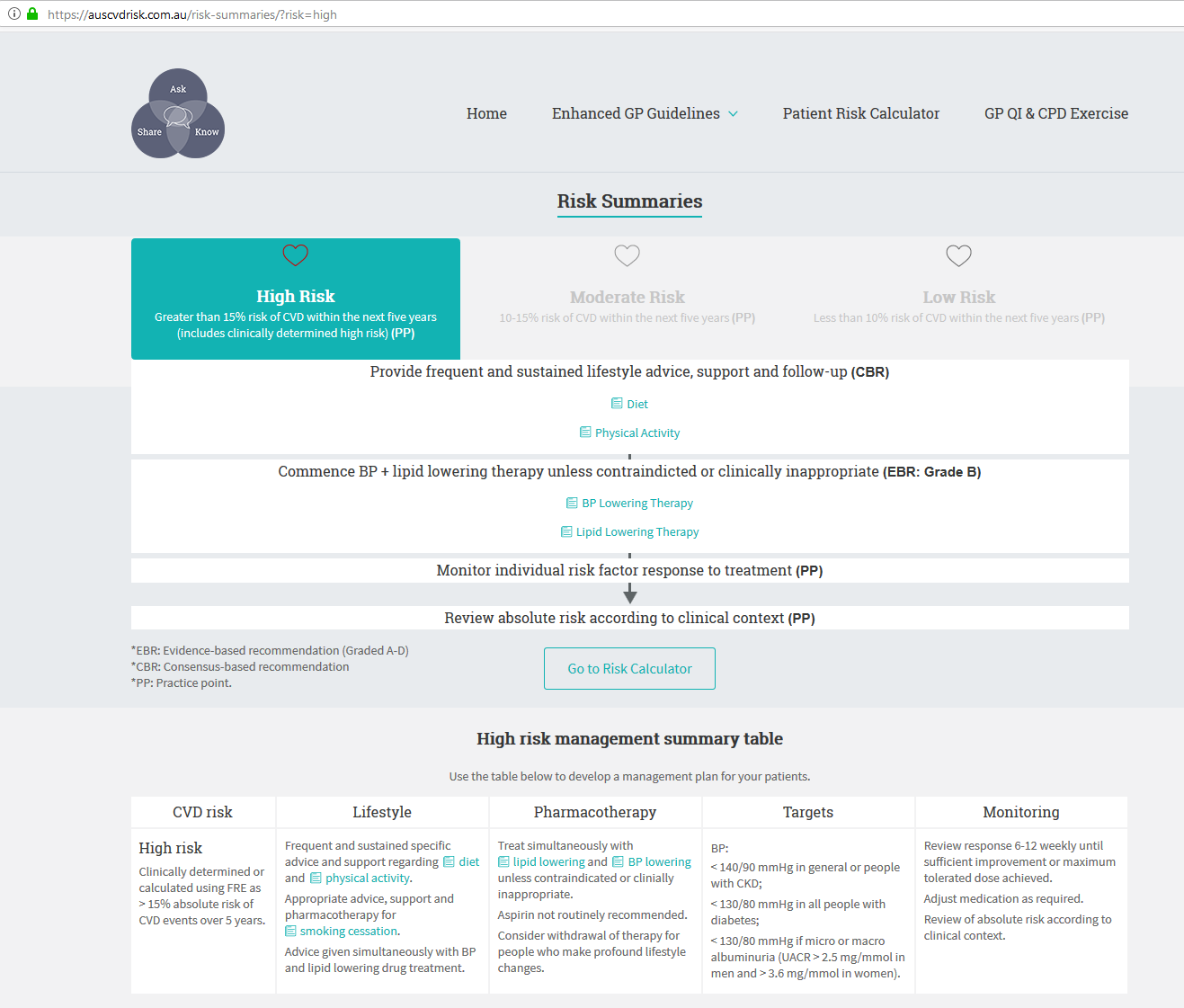


1. **Updated evidence summaries**

Rapid reviews were conducted to update the guideline evidence using the most recent high quality studies on medication, lifestyle and supplement intervention effects on CVD events. This involved processes developed and tested in the ASK-GP Centre of Research Excellence (to be published elsewhere), using MAGICapp to summarise the evidence and then editing the results in a new summary document based on GP format preferences. This process resulted in evidence summaries for the benefits and harms of cholesterol medication, blood pressure medication, aspirin, Mediterranean diet, physical activity, multivitamins, antioxidants and omega-3. The effect of smoking was estimated by recalculating absolute risk as a non-smoker. Below is an example of an evidence summary for Mediterranean diet, which can be accessed within the guidelines (see feature 1) or via recommended management links in the risk calculator results (see feature 3).

***Evidence summary: example intervention effect and practical issues for Mediterranean Diet***


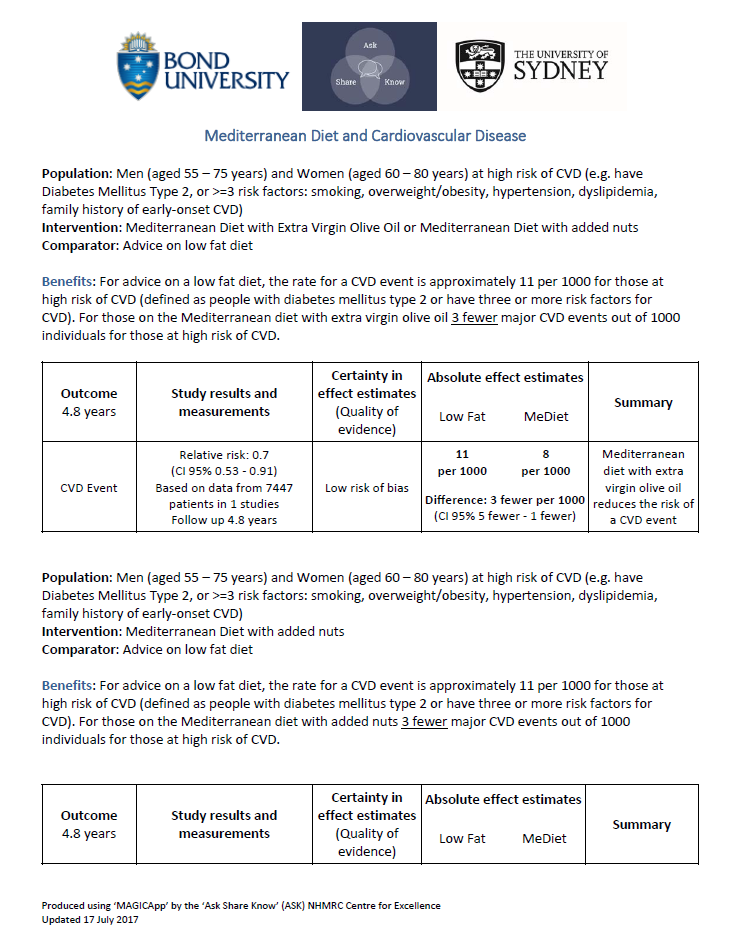


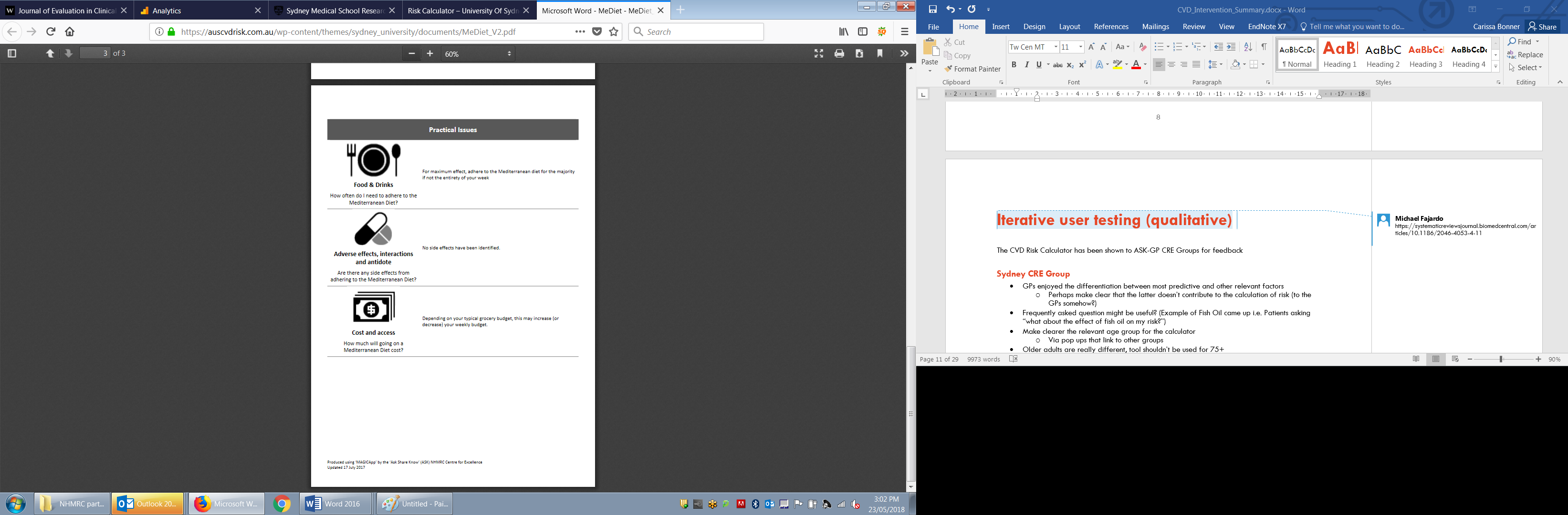


1. **Risk calculator**

The risk calculator was created based on the 5 year Framingham equation, with some adjustments to determine whether patients were deemed to be high risk without the need for an absolute risk calculation. This is based on best practice risk communication principles and feedback from patients on what features they are most interested in (including icon arrays and future risk in 10 years if nothing changes). Below is an example of a high risk result (16% chance of a CVD event in the next 5 years), with management options recommended in the Australian guidelines that link to evidence summaries (see feature 2).

***Risk calculator: example results for risk assessment and management guidelines for a high risk patient***

**
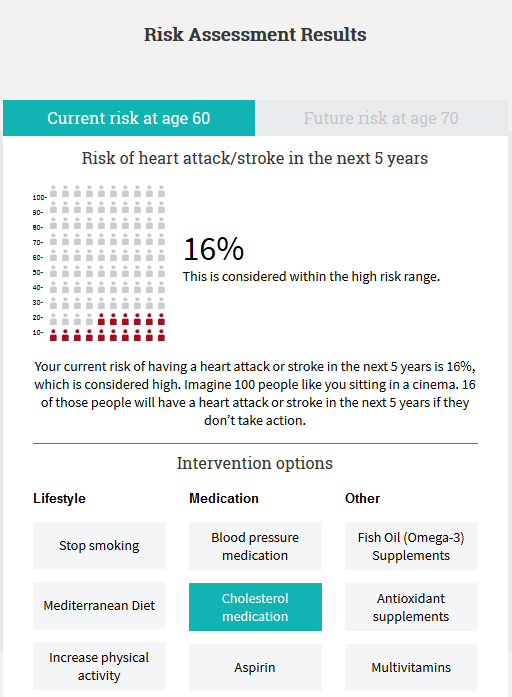
**

**
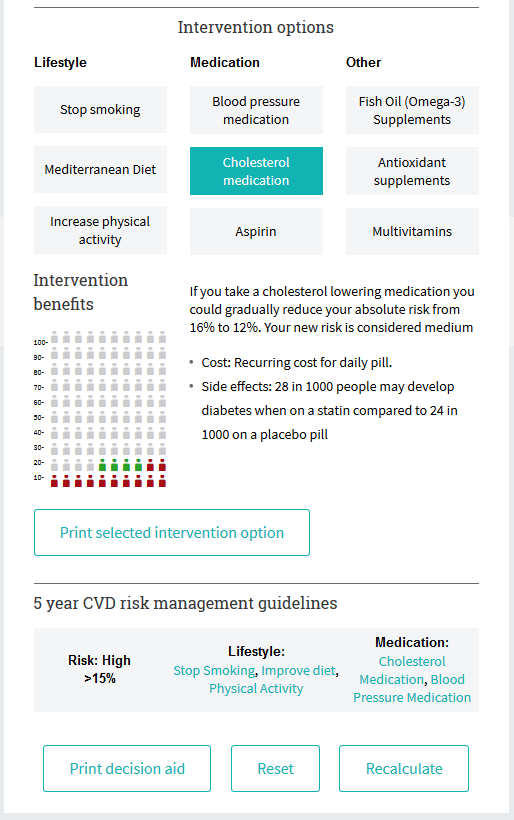
**

1. **Patient decision aid**

An interactive decision aid was developed based on International Patient Decision Aid Standards and patient feedback on preferred features and interventions (including colour-coded icon arrays to show intervention effects, and supplements even though they have no effect on CVD risk). GPs can print a written version of the decision aid with additional information about all management options. The full decision aid includes 9 intervention options and an action plan. The summary version is a 2 page summary of a single intervention, requested by GPs during piloting. Below is an example of the interactive version showing benefits and harms of cholesterol medication for a high risk result of 16% (see feature 3), with the more detailed print version on the next page.

***Patient decision aid: interactive version for cholesterol medication benefits/harms***

**
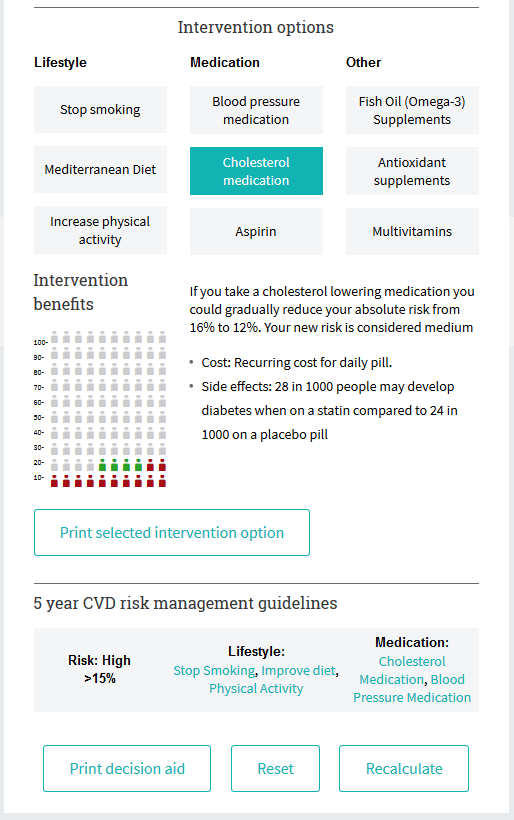
**

***Patient decision aid: print version for cholesterol medication benefits/harms***


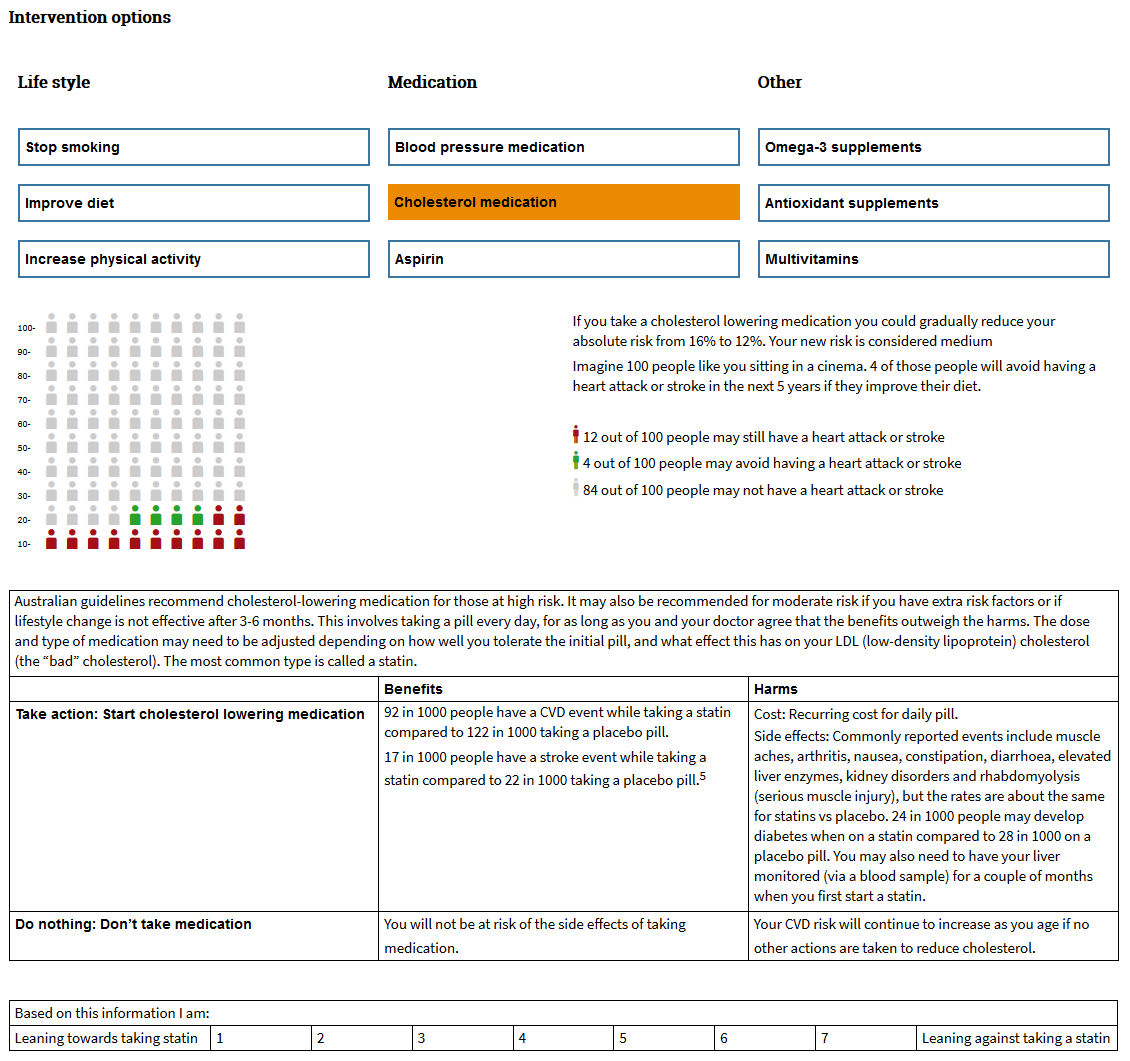


1. **Audit and feedback**

An audit and feedback exercise for GPs was created including 9 case studies identified in our previous research as challenging for GPs, a form to complete for 10 patients, automated feedback comparing current practice to guidelines (including assessment, management and communication) and other GPs, the development of an action plan to be reviewed in 6 months, and optional activities for online peer discussion, anonymous swapped feedback on action plans. The general structure involving an automated report for 10 patients is a common audit and feedback format used in Australia by an organisation called NPS MedicineWise. This format is familiar to Australian GPs for quality improvement and continuing professional development activities. Screenshots of key features are on the following pages.

***Audit & feedback: instructions***


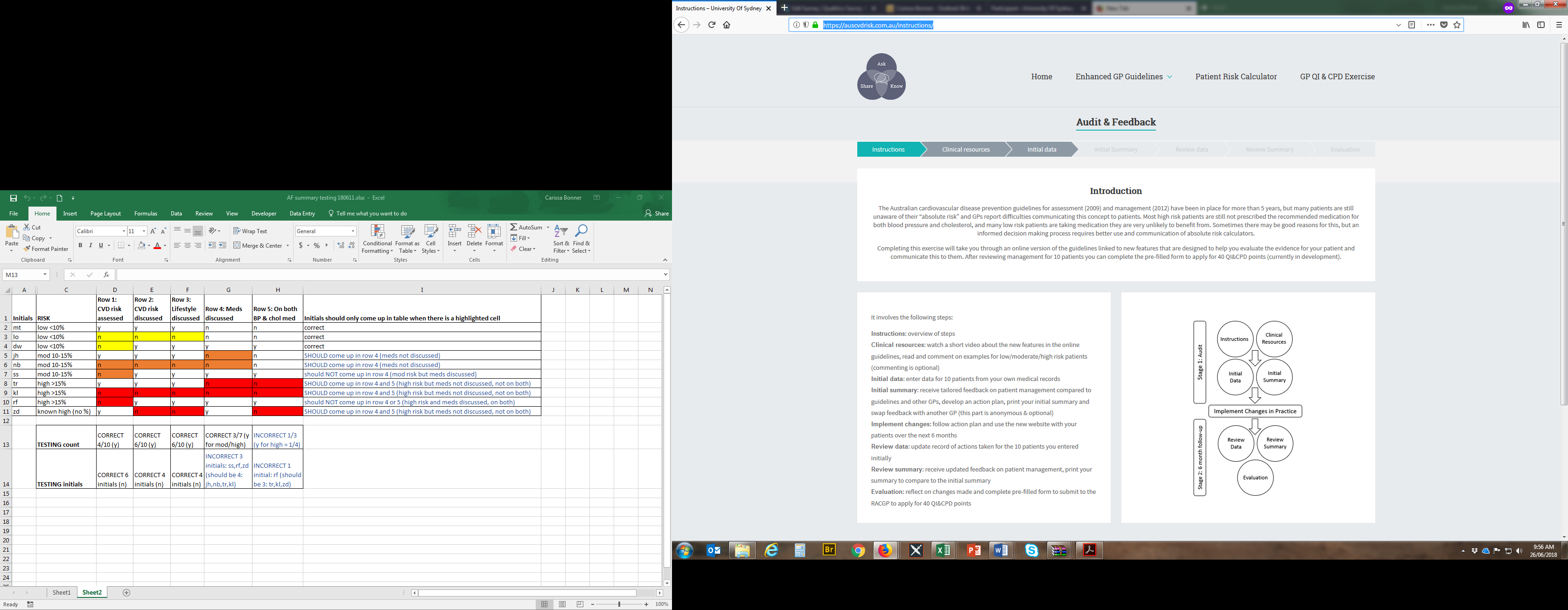


***Audit & feedback: clinical resources including video demonstrating use of risk calculator/decision aid and interactive patient cases with tailored feedback and peer discussion***

***
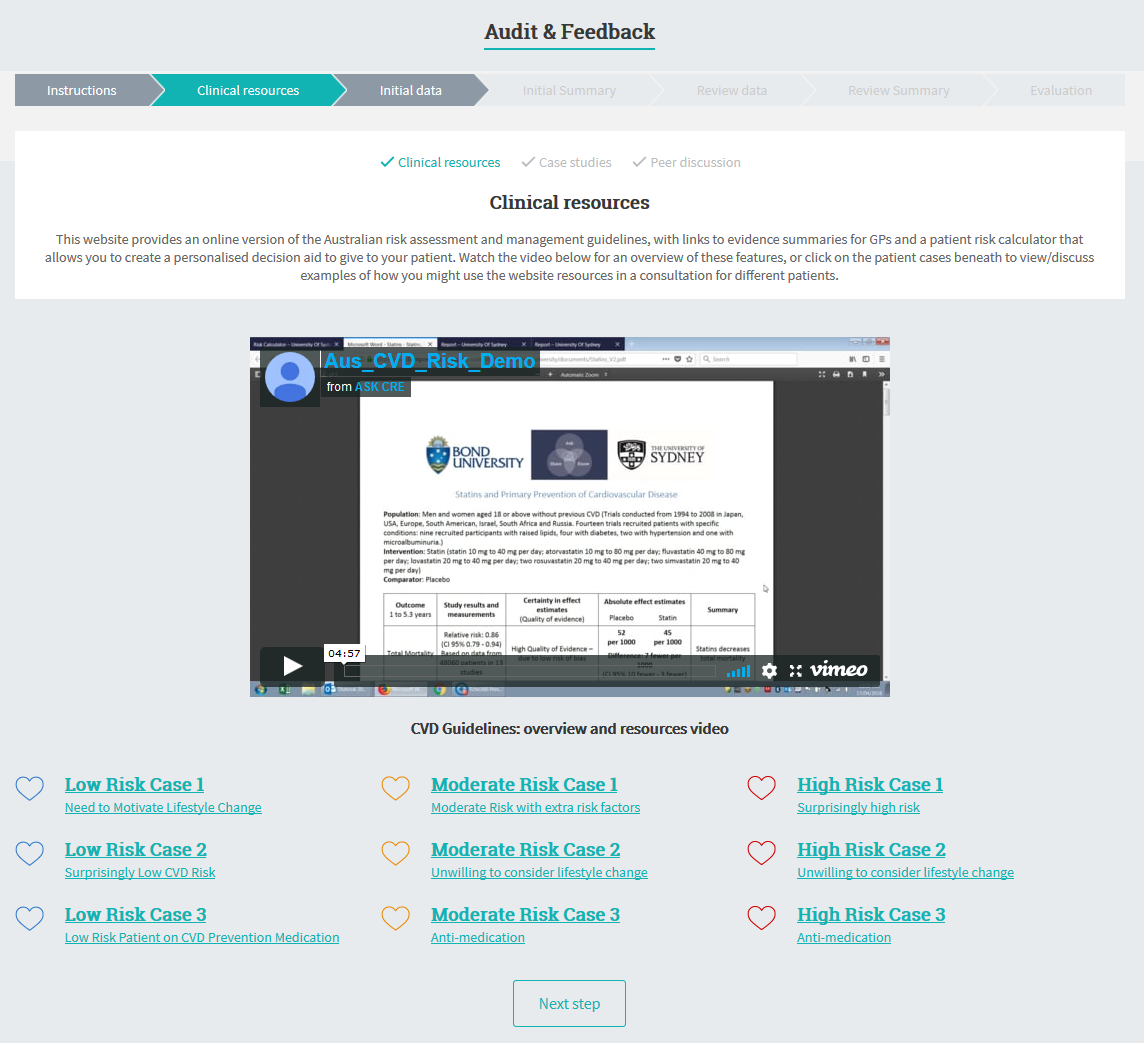
***

***
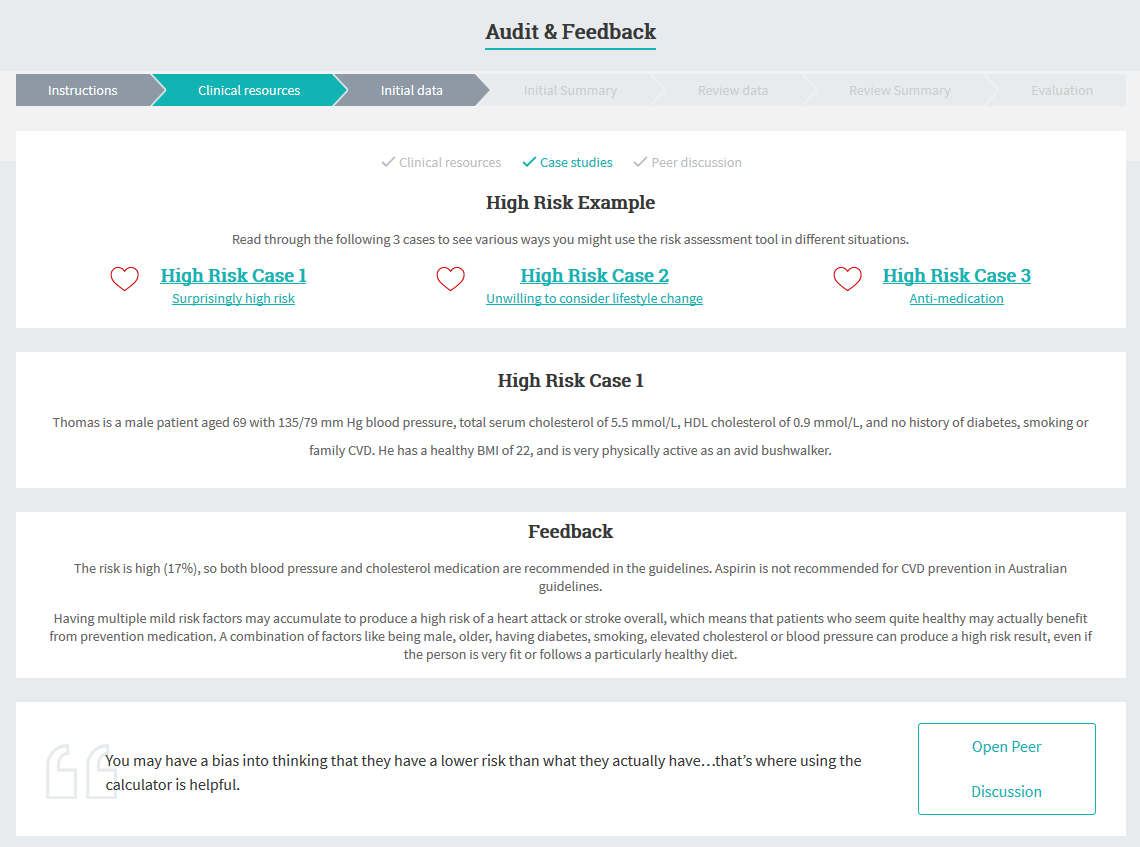
***

***Audit & feedback: automated feedback on current management of 10 patients compared to guidelines and peers, summary of action plan for flagged patients, and option to swap feedback on action plan with another GP anonymously (repeated after 6 months with 3 reminders to implement action plan)***


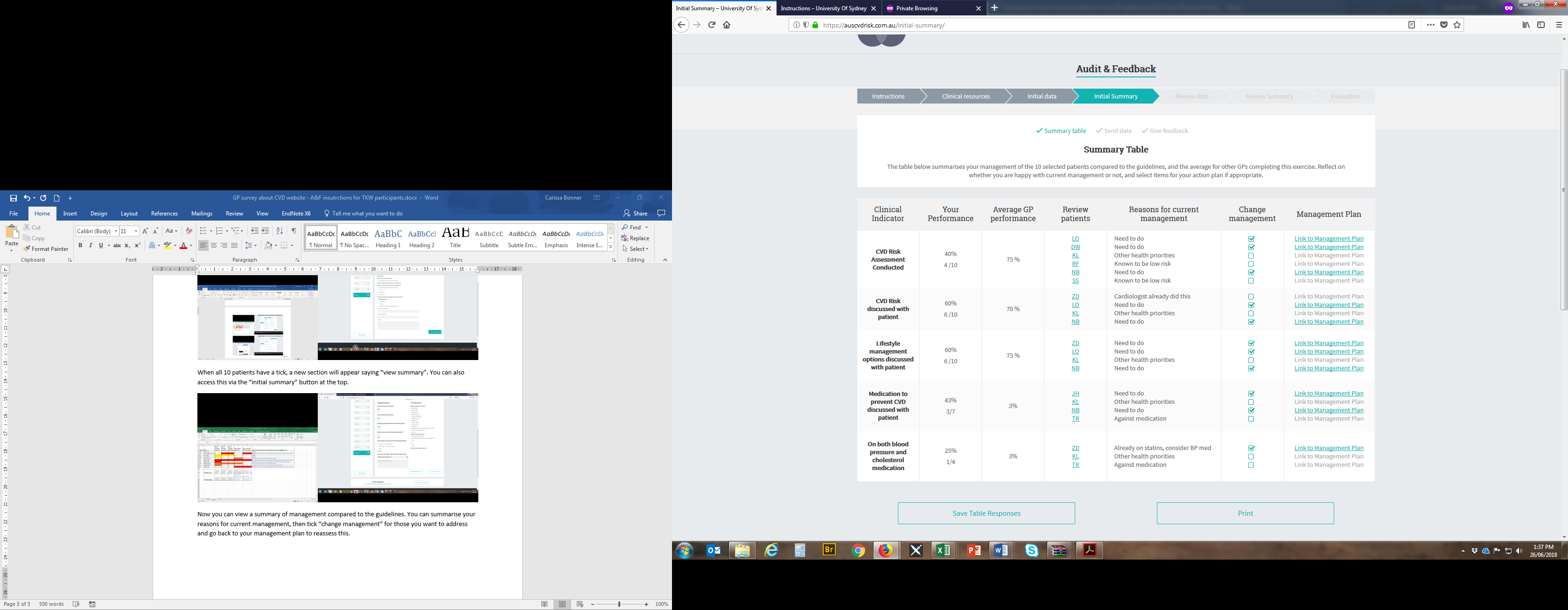


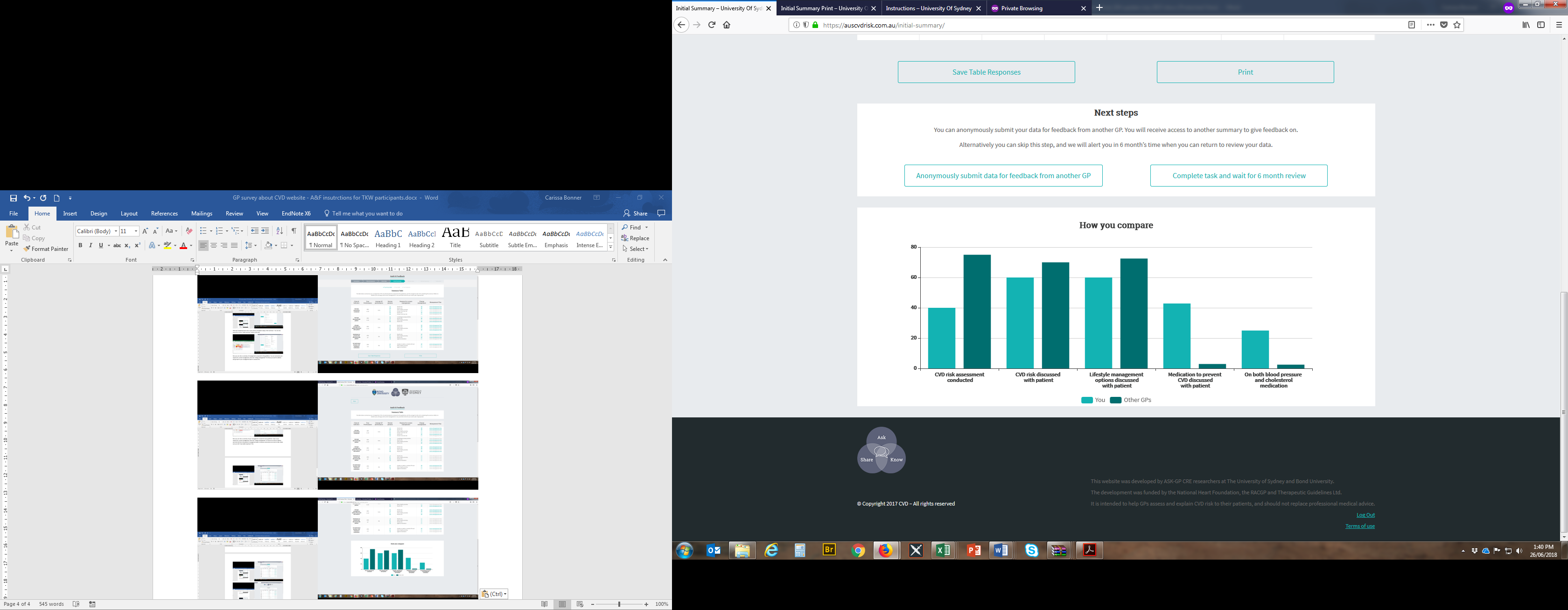

Supplement: Supplementary file 1 — Components of the Australian CVD guidelines intervention. (DOCX 2701 kb) [file 13012_2019_927_MOESM1_ESM.docx]
