# Supplementary material for: Implementing cardiovascular disease prevention guidelines to translate evidence-based medicine and shared decision making into general practice: theory-based intervention development, qualitative piloting and quantitative feasibility
Source: Implement Sci. 2019 Aug 30;14:86. doi: 10.1186/s13012-019-0927-x (PMC6716813; doi:10.1186/s13012-019-0927-x)
Supplement: Supplementary file 2 — GP survey about cardiovascular disease (CVD) prevention guidelines. (PDF 60 kb) [file 13012_2019_927_MOESM2_ESM.pdf]

GP survey about cardiovascular disease (CVD) prevention guidelines

Are you a GP? Do you currently practice in Australia?

If yes, we would like to get your feedback on a new online version of the Australian cardiovascular disease (CVD) prevention guidelines, which includes updated evidence summaries for recommended lifestyle and medication options and patient communication tools

- Answer a 10 minute survey online
- Access a new online version of the Australian CVD risk calculator and associated guidelines
- Answer a 10 minute follow-up survey online after 1 month and claim $40
- Optional: get an access code to complete a related audit & feedback exercise for 10 patients to claim 40 QI&CPD points (by completing and submitting the pre-filled form provided on the website to the RACGP)

View and download/print more information about the study here: GP CVD survey information

○ I confirm I am a GP currently practicing in Australia and consent to participate

○ I do not meet the criteria above and/or do not consent to participate

CVD risk assessment

In the last month, how often did you do the following when you saw patients in the target age for CVD prevention? (1 = never, 7 = always)

	never 1	2	3	4	5	6	always 7
Check smoking status	○	○	○	○	○	○	○
Check blood pressure	○	○	○	○	○	○	○
Check cholesterol	○	○	○	○	○	○	○
Check absolute CVD risk score using a calculator	○	○	○	○	○	○	○

If you did use an absolute CVD risk score, how did you calculate it? (select all that apply)

- ☐ I don’t use them
- ☐ Printed absolute risk chart
- ☐ Risk calculator at www.cvdcheck.org.au
- ☐ Risk calculator in Best Practice software
- ☐ Risk calculator in Medical Director software
- ☐ Clinical judgment to estimate absolute risk
- ☐ Clinical judgment to account for extra risk factors not in risk calculator
- ☐ ________ Other (please specify)

Please rate the following statements (1 = strongly disagree, 7 = strongly agree)

	strongly disagree 1	2	3	4	5	6	strongly agree 7
I am confident I could conduct an accurate absolute CVD risk assessment for my patients if I wanted to	○	○	○	○	○	○	○
I am confident I could explain the absolute CVD risk score to my patients if I wanted to	○	○	○	○	○	○	○

Please rate the following statements (1 = easy, 7 = difficult)

	easy 1	2	3	4	5	6	difficult 7
For me to conduct an accurate absolute CVD risk assessment for my patients is:	○	○	○	○	○	○	○
For me to explain the absolute CVD risk score to my patients is:	○	○	○	○	○	○	○

CVD risk management guidelines

What is the target age group for CVD risk assessment in the general

population?

What is the target age group for CVD risk assessment for Aboriginal and Torres Strait Islanders?

Where have you seen the 2012 Australian guidelines on CVD risk management?

- ☐ I haven’t read them
- ☐ Printed version
- ☐ NVDPA website
- ☐ Heart Foundation website
- ☐ RACGP website
- ☐ Therapeutic Guidelines
- ☐ Professional development activity (please specify)
- ☐ Other (please specify)

What is the recommended 5 year absolute CVD risk threshold for starting preventive medication?

What is the role of the following CVD risk factors in CVD prevention guidelines?

	Determine whether patient is known to be high risk	Calculate patient's 5 year absolute risk of CVD event	Decide on best management approach
Diabetes	☐	☐	☐
Diet	☐	☐	☐
Sex	☐	☐	☐
Familial hypercholesterolaemia	☐	☐	☐
Triglycerides	☐	☐	☐
Waist circumference	☐	☐	☐
	Determine whether patient is known to be high risk	Calculate patient's 5 year absolute risk of CVD event	Decide on best management approach
Stress	☐	☐	☐
Experience of CVD event	☐	☐	☐
Physical activity	☐	☐	☐
Ethnicity	☐	☐	☐
Smoking	☐	☐	☐
Alcohol	☐	☐	☐
	Determine whether patient is known to be high risk	Calculate patient's 5 year absolute risk of CVD event	Decide on best management approach
Age	☐	☐	☐
Cholesterol	☐	☐	☐
Socio-economic status	☐	☐	☐

Select a case to trial with new online guidelines

Please select one of the cases below to try with the new online risk calculator

- Mary is a female patient aged 60 with 130/83 mm Hg blood pressure, total serum cholesterol of 6.4 mmol/L, HDL cholesterol of 1.2 mmol/L with no history of diabetes, family CVD or smoking. She is overweight with a BMI of 29 but hasn't been very motivated to change her diet or lose weight.
- David is a male patient aged 46 with 110/80 mm Hg blood pressure, total serum cholesterol of 6.5 mmol/L and HDL cholesterol of 1.2 mmol/L before another GP put him on statins, with no history of diabetes or family CVD. He has a healthy BMI of 23 and smokes occasionally when stressed at work.
- Robert is a male patient aged 66 with 145/80 mm Hg blood pressure, total serum cholesterol of 7.3 mmol/L, HDL cholesterol of 1.1 mmol/L with no history of diabetes, smoking or family CVD. He has a BMI of 27.5 and doesn't like the idea of any medical intervention.
- Susan is a female patient aged 47 with 159/83 mm Hg blood pressure, total serum cholesterol of 6.3 mmol/L, HDL cholesterol of 0.9 mmol/L, with no history of diabetes or family CVD. She is overweight with a BMI of 27, smokes 1 cigarette a day and is not willing to take drugs because blood pressure medication made her get vertigo in the past, and she has heard bad media reports about statins.
- Karen is a female patient aged 55 with 159/83 mm Hg blood pressure, total serum cholesterol of 7.4 mmol/L, HDL cholesterol of 1.1 mmol/L with no history of diabetes or family CVD. She smokes a pack of cigarettes a day and has given up trying to quit, and is overweight with a BMI of 27.
- Linda is a female patient aged 51 with 114/79 mm Hg blood pressure, total serum cholesterol of 7.0 mmol/L, HDL cholesterol of 1.0 mmol/L with no history of diabetes, familial hypercholesterolaemia, family CVD or smoking. She is very overweight with a BMI of 32.
- James is a male patient aged 61 with 120/81 mm Hg blood pressure, total serum cholesterol of 6.9 mmol/L, HDL cholesterol of 1.5 mmol/L with no history of diabetes or family CVD. He is overweight with a BMI of 28, smokes socially on the weekend, and identifies as Aboriginal.
- Nancy is a female patient aged 65 with 125/80 mm Hg blood pressure, total serum cholesterol of 5.9 mmol/L, HDL cholesterol of 1.1 mmol/L with no history of diabetes or family CVD. She smokes a pack of cigarettes a week and is very overweight with a BMI of 30, but says it's too hard to change her lifestyle.
- Thomas is a male patient aged 69 with 135/79 mm Hg blood pressure, total serum cholesterol of 5.5 mmol/L, HDL cholesterol of 0.9 mmol/L, and no history of diabetes, smoking or family CVD. He has a healthy BMI of 22, and is very physically active as an avid bushwalker.

For this case, what CVD risk category do you think they are in?

- Low risk
- Moderate risk
- High risk

For this case, how likely would you be to prescribe preventive medication to this person?

- Very unlikely
- Unlikely
- Neutral
- Likely
- Very likely

For this case, what do you think their 5 year absolute CVD risk will be?

Results from new online guidelines

For the case you just selected, copy-paste the following link in a new browser window, save the URL to use over the next month, and enter the risk factors into the calculator:

http://auscvdrisk.com.au/risk-calculator

Then answer the following questions.

- » Mary is a female patient aged 60 with 130/83 mm Hg blood pressure, total serum cholesterol of 6.4 mmol/L, HDL cholesterol of 1.2 mmol/L with no history of diabetes, family CVD or smoking. She is overweight with a BMI of 29 but hasn't been very motivated to change her diet or lose weight.
- » Linda is a female patient aged 51 with 114/79 mm Hg blood pressure, total serum cholesterol of 7.0 mmol/L, HDL cholesterol of 1.0 mmol/L with no history of diabetes, familial hypercholesterolaemia, family CVD or smoking. She is very overweight with a BMI of 32.
- » David is a male patient aged 46 with 110/80 mm Hg blood pressure, total serum cholesterol of 6.5 mmol/L and HDL cholesterol of 1.2 mmol/L before another GP put him on statins, with no history of diabetes or family CVD. He has a healthy BMI of 23 and smokes occasionally when stressed at work.
- » James is a male patient aged 61 with 120/81 mm Hg blood pressure, total serum cholesterol of 6.9 mmol/L, HDL cholesterol of 1.5 mmol/L with no history of diabetes or family CVD. He is overweight with a BMI of 28, smokes socially on the weekend, and identifies as Aboriginal.
- » Nancy is a female patient aged 65 with 125/80 mm Hg blood pressure, total serum cholesterol of 5.9 mmol/L, HDL cholesterol of 1.1 mmol/L with no history of diabetes or family CVD. She smokes a pack of cigarettes a week and is very overweight with a BMI of 30, but says it's too hard to change her lifestyle.
- » Susan is a female patient aged 47 with 159/83 mm Hg blood pressure, total serum cholesterol of 6.3 mmol/L, HDL cholesterol of 0.9 mmol/L, with no history of diabetes or family CVD. She is overweight with a BMI of 27, smokes 1 cigarette a day and is not willing to take drugs because blood pressure medication made her get vertigo in the past, and she has heard bad media reports about statins.
- » Thomas is a male patient aged 69 with 135/79 mm Hg blood pressure, total serum cholesterol of 5.5 mmol/L, HDL cholesterol of 0.9 mmol/L, and no history of diabetes, smoking or family CVD. He has a healthy BMI of 22, and is very physically active as an avid bushwalker.
- » Karen is a female patient aged 55 with 159/83 mm Hg blood pressure, total serum cholesterol of 7.4 mmol/L, HDL cholesterol of 1.1 mmol/L with no history of diabetes or family CVD. She smokes a pack of cigarettes a day and has given up trying to quit, and is overweight with a BMI of 27.
- » Robert is a male patient aged 66 with 145/80 mm Hg blood pressure, total serum cholesterol of 7.3 mmol/L, HDL cholesterol of 1.1 mmol/L with no history of diabetes, smoking or family CVD. He has a BMI of 27.5 and doesn't like the

What is the current absolute risk result?

What is the CVD risk category?

- Low risk
- Moderate risk
- High risk

What are the recommended management options?

- Increase physical activity
- Stop smoking
- Cholesterol medication
- Change diet
- Aspirin
- Omega 3 / fish oil supplements
- Antioxidants
- Blood pressure medication
- Multivitamin supplements

What does the CVD risk result reduce to when you select one of these options?

Blood pressure medication	
Aspirin	
Cholesterol medication	
Omega 3 / fish oil	
Multivitamins	
Antioxidants	
Mediterranean diet	
Physical activity	
Stop smoking	

What is the patient's future absolute risk result in 10 years if nothing changes?

Block 5

Below is a list of website features. Which sections of the website did you look at, and which will you use in the next month?

	Already looked at this	Will use in the next month
Risk calculator for current risk	☐	☐
Button to show future risk (in 10 years)	☐	☐
Buttons showing intervention effects on risk result (medication, lifestyle, supplements)	☐	☐
View selected intervention (2 page summary for 1 intervention option)	☐	☐

	Already looked at this	Will use in the next month
View full decision aid (report on patient's risk results + all 9 intervention options)	☐	☐
Risk assessment guidelines	☐	☐
Low risk guidelines	☐	☐
Moderate risk guidelines	☐	☐

	Already looked at this	Will use in the next month
High risk guidelines	☐	☐
Links to evidence summaries for intervention [illegible]	☐	☐

What would need to change for you to use the website more?

How could we improve the website?

Final questions

What is your gender?

- Male
- Female
- Other/prefer not to say

In what year were you born?

In what year did you qualify as a doctor?

How many GPs work in your practice?

In which state/territory do you currently practice?

- ☐ ACT
- ☐ New South Wales
- ☐ Northern Territory
- ☐ Queensland
- ☐ Tasmania
- ☐ Victoria
- ☐ Western Australia
- ☐ ________ Postcode

Block 7

Thank you for participating. We will contact you again in 1 month to complete a shorter follow-up survey. Please save the URL below if you have not already, and use the new risk calculator with any of your patients over the next month so we can get your feedback on how to improve it.

http://auscvdrisk.com.au/risk-calculator

If you would like to complete the audit and feedback exercise of 10 patients to receive 40 QI&CPD points from the RACGP, the recruitment company will provide you with an access code after you have completed the second survey.

Powered by Qualtrics
